# Mendelian randomization analysis rules out disylipidaemia as colorectal cancer cause

**DOI:** 10.1038/s41598-019-49880-w

**Published:** 2019-09-16

**Authors:** Gemma Ibáñez-Sanz, Anna Díez-Villanueva, Marina Riera-Ponsati, Tania Fernández-Villa, Pablo Fernández Navarro, Mariona Bustamante, Javier Llorca, Pilar Amiano, Nieves Ascunce, Guillermo Fernández-Tardón, Inmaculada Salcedo Bellido, Dolores Salas, Rocío Capelo Álvarez, Marta Crous-Bou, Luis Ortega-Valín, Beatriz Pérez-Gómez, Gemma Castaño-Vinyals, Camilo Palazuelos, Jone M. Altzibar, Eva Ardanaz, Adonina Tardón, José Juan Jiménez Moleón, Valle Olmos Juste, Nuria Aragonés, Marina Pollán, Manolis Kogevinas, Victor Moreno

**Affiliations:** 1 Unit of Biomarkers and Susceptibility, Oncology Data Analytics Program, Catalan Institute of Oncology (ICO) and ONCOBELL Program, Bellvitge Biomedical Research Institute (IDIBELL), L’Hospitalet de Llobregat, Spain; 2 Gastroenterology Department, Bellvitge University Hospital, L’Hospitalet de Llobregat, Spain; 3 CIBER Epidemiología y Salud Pública (CIBERESP), Madrid, Spain; 4 Grupo de Investigación en Interacciones Gen-Ambiente y Salud. Instituto de Biomedicina (IBIOMED). University of León, León, Spain; 5 Environmental and Cancer Epidemiology Department, National Center of Epidemiology - Instituto de Salud Carlos III, Madrid, Spain; 6 Oncology and Hematology Area, IIS Puerta de Hierro, Cancer Epidemiology Research Group, Madrid, Spain; 7 ISGlobal, Barcelona, Spain; 8 IMIM (Hospital del Mar Medical Research Institute), Barcelona, Spain; 9 University of Pompeu Fabra, Barcelona, Spain; 10 University of Cantabria – IDIVAL, Santander, Spain; 11 Public Health Division of Gipuzkoa, Biodonostia Research Institute, San Sebastian, Spain; 12 Navarra Public Health Institute, Pamplona, Spain; 13 IdiSNA, Navarra Institute for Health Research, Pamplona, Spain; 14 University Institute of Oncology of Asturias (IUOPA), Universidad de Oviedo, Oviedo, Spain; 15 Instituto de Investigación Biosanitaria de Granada (ibs.GRANADA), Hospitales Universitarios de Granada/ University of Granada, Granada, Spain; 16 Department of Preventive Medicine and Public Health, Faculty of Medicine, University of Granada, Granada, Spain; 17 Cancer and Public Health Area, FISABIO - Public Health, Valencia, Spain; 18 General Directorate Public Health, Valencia, Spain; 19 Centro de Investigación en Recursos Naturales, Salud, y Medio Ambiente (RENSMA), University of Huelva, Huelva, Spain; 20 Barcelonaβeta Brain Research Center - Pasqual Maragall Foundation, Barcelona, Spain; 21 CIBER Fragilidad y Envejecimiento Saludable (CIBERFES), Madrid, Spain; 22 Department of Epidemiology, Harvard T. H. Chan School of Public Health, Boston, MA USA; 23 Department of Pharmacy, Complejo Asistencial Universitario de León, León, Spain; 24 Department of General and Gastrointestinal Surgery, Complejo Asistencial Universitario de León, León, Spain; 25 Dirección General de Salud Pública, Consejería de Sanidad de la Comunidad de Madrid, Madrid, Spain; 26 Department of Clinical Sciences, Faculty of Medicine and Health Sciences, University of Barcelona, Barcelona, Spain

**Keywords:** Cancer epidemiology, Cancer prevention

## Abstract

Dyslipidemia and statin use have been associated with colorectal cancer (CRC), but prospective studies have shown mixed results. We aimed to determine whether dyslipidemia is causally linked to CRC risk using a Mendelian randomization approach and to explore the association of statins with CRC. A case-control study was performed including 1336 CRC cases and 2744 controls (MCC-Spain). Subjects were administered an epidemiological questionnaire and were genotyped with an array which included polymorphisms associated with blood lipids levels, selected to avoid pleiotropy. Four genetic lipid scores specific for triglycerides (TG), high density lipoprotein cholesterol (HDL), low density lipoprotein cholesterol (LDL), or total cholesterol (TC) were created as the count of risk alleles. The genetic lipid scores were not associated with CRC. The ORs per 10 risk alleles, were for TG 0.91 (95%CI: 0.72–1.16, p = 0.44), for HDL 1.14 (95%CI: 0.95–1.37, p = 0.16), for LDL 0.97 (95%CI: 0.81–1.16, p = 0.73), and for TC 0.98 (95%CI: 0.84–1.17, p = 0.88). The LDL and TC genetic risk scores were associated with statin use, but not the HDL or TG. Statin use, overall, was a non-significant protective factor for CRC (OR 0.84; 95%CI: 0.70–1.01, p = 0.060), but lipophilic statins were associated with a CRC risk reduction (OR 0.78; 95%CI 0.66–0.96, p = 0.018). Using the Mendelian randomization approach, our study does not support the hypothesis that lipid levels are associated with the risk of CRC. This study does not rule out, however, a possible protective effect of statins in CRC by a mechanism unrelated to lipid levels.

## Introduction

Colorectal cancer (CRC) has been associated with both genetic and environmental risk factors such as diet^1,2^, alcohol^3^, smoking^4^, physical activity^5^ and metabolic syndrome^6,7^. Metabolic syndrome is a cluster of important cardiovascular risk factors: high fasting glucose, abdominal obesity, high triglycerides (TG), reduced high density lipoprotein cholesterol (HDL) and high blood pressure. As one of the components, dyslipidemia has been thought to have an important role in inflammatory pathways, oxidative stress and insulin resistance, which could contribute to the pathogenesis of cancer. However, findings from prospective studies that have examined the association between serum dyslipidemia (TG, HDL, low density lipoprotein cholesterol (LDL) or total cholesterol (TC)) and colorectal neoplasia have been inconsistent^6,8–11^. It is unknown whether lipids and lipoproteins cause cancer or are intermediate or correlated factors within carcinogenic pathways. The Mendelian randomization approach can be used to establish a causal relationship between dyslipidemia and CRC. Mendelian randomization studies use the distribution of alleles in the population to simulate randomized assignment to lower or higher lipids. A previous Mendelian randomization analysis assessing the causality of dyslipidemia and CRC has been published before^11^. It reported an association between a genetic score for TC and the risk of CRC (OR per unit SD increase = 1.46; 95% CI: 1.20–1.79) but no significant association was found for LDL, HDL or TG. While the association of TC was strong, the interpretation of the results is not straightforward, since TC is the sum of LDL and HDL cholesterol, fractions that have been reported to have opposite effects regarding CRC and may explain the controversial findings of studies that have analyzed the association between high levels of serum TC and CRC risk^8,9,12^. A causal effect of lipids regarding CRC risk should have a clear mechanistic interpretation. Being TC essentially the sum of HLD and LDL, genetic instruments for TC are correlated either with LDL, HDL or both, which violates the pleiotropy assumption of Mendelian randomization studies.

Epidemiological studies on dyslipidemia and CRC risk could be confounded by 3-Hydroxy-3-methylglutaryl-coenzyme A reductase inhibitors (statins) use, which might also have a protective effect on CRC. It is unclear whether it is statin use or dyslipidemia that prompted statin use, which may be associated with CRC. Indeed, a large number of epidemiological studies have examined the effect of statins on CRC risk, with often inconsistent results^13,14^. It has been suggested that pharmacogenetic variation or specific type of statin, which influence the effect on lipid levels, might also modify the statin-CRC risk association^13–15^.

In this study, we conducted a Mendelian randomization study to evaluate the relationship between CRC and a genetic risk score, derived from 119 genetic variants associated with blood concentrations of TG, HDL and LDL in GWAS studies. Moreover, we wanted to explore the effect of statins use on CRC.

## Materials and Methods

### Study population

A detailed description of the MCC-Spain case-control study has been provided elsewhere^16^. Briefly, 10,183 subjects aged 20–85 years were enrolled in Spanish hospitals and primary care centers between 2008 and 2013. Eligible subjects included histological confirmed incident cases of CRC (n = 2,171). Both cases and controls were free of personal CRC history. For the present study we only have included a subset of 1,336 CRC cases and 2,744 controls that had genotype data. These subjects were selected from the complete study using a stratified random sampling strategy to maintain the distribution of cases and controls among participating centers.

### Data collection

A structured computerized epidemiological questionnaire was administered by trained personnel in a face-to-face interview. Subjects also filled in a semi-quantitative food frequency questionnaire, and blood samples and anthropometric data were obtained following the study protocol. This study did not collect or measured blood lipid levels for cases and controls.

The variables that were analyzed were those that could be related with CRC. We also wanted to explore variables included in the definition of metabolic syndrome. So the variables considered for analyses were: level of education, family history of CRC (none versus first or second or third-degree); cigarette smoking (ever, never); average alcohol consumption between ages 30 and 40 (in standard units of alcohol, SUA, categorized into low-risk and high-risk consumption: >4 SUA/day in men and >2 SUA/day in women)^17^; diabetes (with only diet or using anti-diabetic drugs; hypertension (with only diet or antihypertensive treatment); body mass index (BMI), calculated with the weight reported at age 45, which was categorized according to World Health Organization criteria: underweight, normal weight, and overweight (<30 kg/m^2^) versus obese (≥30 kg/m^2^); abdominal obesity (calculated at the inclusion date) was defined according to World Health Organization criteria as a waist–hip ratio ≥ 0.90 cm for males and ≥0.85 cm for females; average physical exercise, determined using self-reported leisure-time activity performed in the past 10 years and used to estimate the Metabolic Equivalent of Task (MET) per hour per week, calculated using the Ainsworth’s compendium of physical activities^18^, and categorized as no physical activity in their leisure time (0 MET), and any physical activity in their spare time (>0 MET); vegetables, classified as low or high intake using 200 g/day as cut-off; red meat consumption including cured meat, and processed meat. High intake of red meat was considered eating ≥65 g/day.

The location of the CRC was defined according to its anatomic distribution: proximal colon (colon above the level of the splenic flexure including it), distal (descending colon, sigmoid colon), and rectum.

All procedures performed in studies involving human participants were in accordance with the ethical standards of the institutional and/or national research committee and with the 1964 Helsinki declaration. The protocol of MCC-Spain was approved by each of the Ethics Committees of the participating institutions. The specific study reported here was approved by the Bellvitge Hospital Ethics Committee with reference PR 149/08. Written informed consent was obtained from all individual participants included in the study.

### Drug exposure

Use of prescription drugs was obtained through face-to-face interviews administered by trained personnel, mainly by indication. Information was coded following the Anatomical Therapeutic Chemical-ATC code to assess individual exposure to different drugs. All drugs prescribed to the patients were recorded, but only statins, aspirin (acetyl salicylic acid, ASA) or other nonsteroidal anti-inflammatory drugs (NSAIDs) were considered for this study. Statins were classified as lipophilic (atorvastatin, fluvastatin, lovastatin, simvastatin) or hydrophilic (pravastatin and rosuvastatin) and, by effectiveness in lowering LDL cholesterol levels, as low-potency (fluvastatin, lovastatin, pravastatin, simvastatin) and high-potency (atorvastatin and rosuvastatin)^19^. Moreover, statins users were considered regular if they started consuming these drugs at least one year before. Given that ASA or NSAIDs can be used sporadically, regular users were defined as consuming ≥1 time/day for at least one year.

### Genotyping and elaboration of genetic lipid scores

The Infinium Human Exome BeadChip (Illumina, San Diego, USA) array was used to genotype >200,000 coding markers plus 5000 additional custom single nucleotide polymorphisms (SNP) selected from previous genome-wide association studies (GWAS) or in genes of interest for cancer. SNPs associated with specific blood lipid levels (LDL, HDL, TG) in GWAS were identified through the GWAS catalogue^20^ and downloaded with the MRInstruments R package (https://github.com/MRCIEU/MRInstruments). Retrieved SNPs were filtered according to these criteria: associated at genome-wide significance (p ≤ 5.0 × 10^−8^); restricted to Caucasian population; the allele associated with increased lipid level should be identified; only one SNP per chromosomal region (the most statistically significant SNP was selected when linkage disequilibrium was r^2^ > 0.2). To minimize pleiotropic effects, SNPs associated with more than one lipid trait (except for TC, which could share SNPs of HDL and LDL) or to other traits potentially related to CRC (alcohol, BMI, diabetes, inflammation) were excluded. SNP rs174546, which encodes for a protein that is a member of the fatty acid desaturase (FADS), was the only polymorphism that was associated with the three lipid pathways (an increase of LDL and HDL levels, but a decrease of TG), but many other SNPs were associated with more than one lipid trait. So, only SNPs exclusively associated at genome-wide significance with one lipid trait (either TG, HDL or LDL, but not more than 1) were chosen for each genetic risk score to make them as specific to one trait as possible (as in Holmes *et al*.^21^). To study total cholesterol (TC), since it was the sum of LDL and HDL, the SNPs selected were not restricted regarding pleiotropy^22^ between LDL and HDL (some selected SNPs may be related to both LDL and HDL). We consider the results for TC difficult to interpret, since LDL and HDL may have opposite effects. However, we provide the analysis for completeness and to compare the results with other studies.

Since the effects of each individual locus identified through GWAS are small, lipid genetic scores (LGS) were created as the joint additive effect of the selected SNPs risk alleles to increase the power of the Mendelian randomization analysis^23^. We created four LGS: one for each lipid exposure of interest. Our exome array included 22 SNPs associated with levels of TG, 36 with HDL, 39 with LDL and 49 with levels of TC, which are shown in Supplementary Table 1.

In all LGS, risk alleles were those associated with increase of TG, LDL, HDL or TC levels. Each SNP was coded according to the number of copies of the allele associated with dyslipidemia (0, 1, and 2) and each LGS was created as the total sum of alleles across the specific SNPs for each lipid. To simplify the analyses, and because the effect size was not always reported or was in different units, an equal weight was assigned to all SNPs, though it is known that some SNPs show stronger associations with lipid levels than others.

### Statistical analysis

Hardy-Weinberg equilibrium was tested among controls, and no SNP showed deviation with p < 0.001. The sum of allele counts for each LGS was compared between cases and controls with linear models. Odds ratios (OR) and 95% confidence intervals (95% CI) were also calculated from logistic regression models. When referring to genetic scores, all ORs were scaled to express the risk per 10 alleles (OR_10_). A study design adjustment score (SDAS) was created to reduce bias related to differences in case and control selection frequencies. All the analyses were adjusted for this SDAS that included age, sex, recruiting center, level of education and the three first principal components of genetic ancestry obtained from ancestry informative markers included in the genotyping array. The SDAS also included the interactions between age and sex, and region and sex. In addition to the SDAS, which was always considered, multivariate-adjusted models to assess the net effect of statins also included as potential confounders variables that were associated both with CRC (Table 1) and statins (Supplementary Table 2 shows the association of statin use with other variables among controls). To account for multiple comparisons when individual SNPs were analyzed, Bonferroni significance threshold were calculated (Supplementary Table 1). The SNPassoc^24^ package from R statistical software (R Foundation for Statistical Computing, Vienna, Austria) and PLINK^25^ were used for the analyses.

**Table 1:** Characteristics of the MCC-Spain study participants and association of every risk factor with CRC.

Characteristic	Controls (n = 2744)		CRC cases (n = 1336)		Crude OR^a^	95% CI	P-value	Adjusted OR^b^	95% CI	P-value
	n	%	n	%						
Age (years) at index date, median (IQR)	65	(56–72)	68	(60–76)						
Male sex	1469	53.5	865	64.8						
Family history of CRC	333	12.1	292	21.9	2.29	1.90–2.75	<0.0001	2.31	1.91–2.78	<0.0001
Cigarette smoking history	1549	56.5	779	58.3	1.20	1.04–1.38	0.01	1.07	0.93–1.24	0.34
High risk consumption alcohol	427	15.6	300	22.5	1.38	1.16–1.63	0.0002	1.31	1.10–1.56	0.0027
Diabetes mellitus	400	14.6	234	10.6	1.02	0.85–1.23	0.84	1.07	0.85–1.34	0.56
Arterial hypertension	1055	38.5	562	42.1	0.95	0.82–1.09	0.43	0.94	0.82–1.09	0.44
High waist–hip ratio^c^	1875	68.3	1082	81.0	1.34	1.13–1.59	0.0007	1.30	1.09–1.54	0.0032
Obesity (BMI ≥30 kg/m^2^)	188	6.9	142	10.6	1.36	1.07–1.73	0.01	1.29	1.04–1.64	0.043
Physical activity in leisure time	1057	38.5	619	46.3	0.73	0.63–0.84	<0.0001	0.74	0.65–0.80	0.0001
High intake of vegetables (>200 g/day)	846	30.8	345	25.8	0.72	0.62–0.84	<0.0001	0.75	0.64–0.88	0.0004
High intake of red meat (>65 g/day)	1123	40.9	674	50.5	1.38	1.20–1.59	<0.0001	1.30	1.13–1.50	0.0002
Regular ASA users	327	11.9	150	11.2	0.78	0.63–0.97	0.02	0.73	0.59–0.91	0.0060
Regular NSAIDs non-ASA users	422	15.4	122	9.1	0.56	0.45–0.69	<0.0001	0.59	0.47–0.74	<0.0001
Regular statin users	535	19.6	250	18.7	0.81	0.68–0.96	0.02	0.84	0.70–1.01	0.060

ASA: acetylsalicylic acid; BMI: body mass index; MET: Metabolic equivalent of task (MET) per hour per week; NSAID: Nonsteroidal Anti-inflammatory Drugs.

^a^ORs and 95% CI derived from logistic regression models adjusted for the study design factors (age, sex, center and education).

^b^Each variable adjusted for the study design adjustment and potential confounders for statin use (alcohol, waist-hip ratio, physical activity, read meat and ASA or NSAIDs).

^c^≥ 0.90 cm (men); ≥0.85 cm (women).

### Statistical power

We performed a power calculation regarding the association of the LGS with CRC. We used the web application mRNd^26^ (https://cnsgenomics.shinyapps.io/mRnd), and assumed that the proportion of lipid levels variance explained by the LGS was 10%. Our study had 80% power to detect an OR of at least 1.33.

## Results

In this population-based case-control study, as expected, CRC was associated with family history, high alcohol consumption, high waist-hip ratio, physical inactivity, low intake of vegetables, and high intake of red meat (Table 1). Regular AAS or NSAID use was associated with a reduced risk of CRC. Other covariates related with the metabolic syndrome such as diabetes mellitus and arterial hypertension were not associated with CRC.

Statin use was a borderline non-significant protective factor for CRC in the MCC-study in the multivariate analysis (OR = 0.84; 95% CI: 0.70–1.01, p = 0.060, Table 1). A total of 785 patients (535 controls and 250 CRC cases) were current regular users of 3-Hydroxy-3-methylglutaryl-coenzyme A reductase inhibitors. The most frequently used was simvastatin (n = 373, 47.5% of users) followed by atorvastatin (n = 277, 35.3% of users).

Table 2 shows that only lipophilic statins (atorvastatin, fluvastatin, lovastatin, simvastatin), that were the most frequently used, were associated with a CRC risk reduction (OR = 0.79; 95% CI: 0.66–0.96, p = 0.016) but hydrophilic statins (pravastatin and rosuvastatin) did not. No differences were observed regarding lipid lowering potency. We also performed a stratified analysis to determine whether gender influenced the effect of statins on CRC. Statins had a stronger protective effect in men (adjusted OR = 0.80; 95% CI: 0.64–1.00 for men and OR = 0.92; 95% CI: 0.68–1.24 for women), but there was no sex interaction effect (P-value for interaction = 0.43). There was no effect modification when analyzing CRC location.

**Table 2:** Statin use and CRC risk.

		Controls		CRC cases		Adjusted OR^b^	95% CI	P-value
		Statin users	%^a^	Statin users	%^a^			
**Statin characteristics**								
Lipophilicity	Non-users	2209	80.5	1086	81.3	1.00		
	Lipophilic	508	18.5	223	16.7	0.79	0.66–0.96	0.016
	Hydrophilic	26	0.9	26	1.9	1.69	0.94–3.04	0.077
Potency	Non-users	2209	80.5	1086	81.3	1.00		
	Low	346	12.6	154	11.5	0.82	0.66–1.01	0.066
	High	189	6.9	96	7.2	0.88	0.66–1.16	0.38
**Subgroup analysis**								
Gender	Non-users	1087	67.0	382	60.4	1.00		
	Female	188	11.6	89	14.1	0.92	0.68–1.24	0.58
	Male	347	21.4	161	25.4	0.80	0.64–1.00	0.046
Cancer location	Non-users	2209	80.5	626	73.1	1.00		
	Colon	535	19.5	144	16.8	0.86	0.69–1.06	0.16
	Rectum			86	10.0	0.81	0.62–1.06	0.13

^a^% of statin users over total number in subgroup.

^b^ORs and 95% CI derived from logistic regression models adjusted for the study design factors (age, sex, center and education), alcohol, waist-hip ratio, physical activity, red meat, and ASA or NSAID use.

### LDL genetic score and statins use

**Figure 1:**
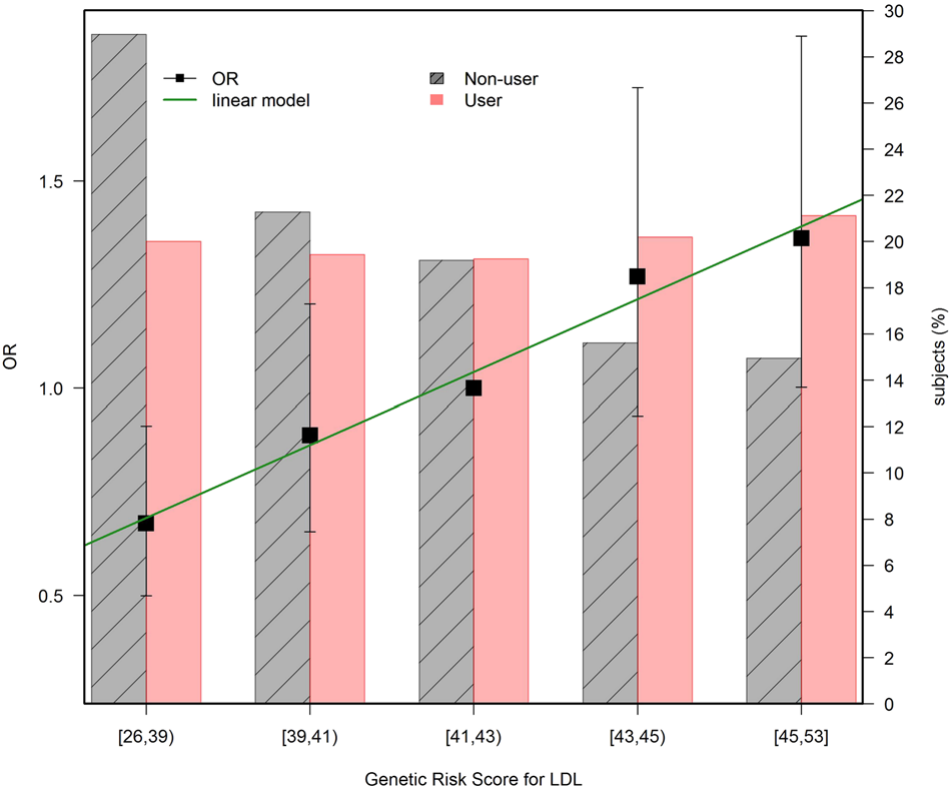
LDL LGS and statin use (Controls only). The black dots follow the left axis scale and correspond to the OR for statin use according to the number of LDL LGS risk alleles. The reference group is the median in the population, or subjects with 41–43 risk alleles. The green line corresponds to a linear model fitted to the ORs, to emphasize the linear relationship that can be interpreted as independent contribution of each SNP to the LDL LGS. Each risk allele increases 7.8% the likelihood of being regular statin user. The bars follow the right axis scale and indicate the proportion of users (red) and non-users (gray) for each allele count grouping.

The LDL and TC LGS were associated with statin consumption among controls (OR_10_ = 2.12, 95% CI: 1.64–2.73, p < 0.0001 for LDL; OR_10_ = 1.67, 95% CI: 1.32–2.11, p < 0.0001 for TG). Figure 1 shows, the increase in odds ratio of being a regular statin user in relation to the number of risk alleles of the LDL LGS. The figure shows that this increase was linear, indicating an independent additive contribution of each SNP to the LDL score. Each risk allele increased a 7.8% the likelihood of being a regular statin user. This association was similar in cases. Though blood lipid levels for the subjects were not collected in the MCC study, this observation supports the validity of the LDL LGS used as an instrumental variable for lifetime exposure to higher LDL levels. HDL and TG LGS were not associated with statin use (OR_10 = _1.10, 95%CI: 0.86–1.42, p = 0.49 for HDL and OR_10 = _1.33, 95%CI: 0.96–1.84, p = 0.092 for TG).

### Association of lipid genetic scores with CRC

None of the SNPs contributing to each of the LGS was associated with CRC when Bonferroni correction was applied (see Supplementary Tables 1–4 for details). Moreover, the distribution of estimated risk effects showed a normal distribution around an average of zero, with some SNPs showing a direct association with CRC while others had an inverse direction, suggesting that the observed effects were likely related to random variation. The most significant SNPs in relation with CRC in our population were rs1748195 for TG, rs7941030 for HDL, rs12916, rs10102164 and rs10102164 for LDL. For TC, the most significant SNPs were rs12916, rs10102164 and rs7941030 which also were related to LDL and HDL, as expected.

**Figure 2:**
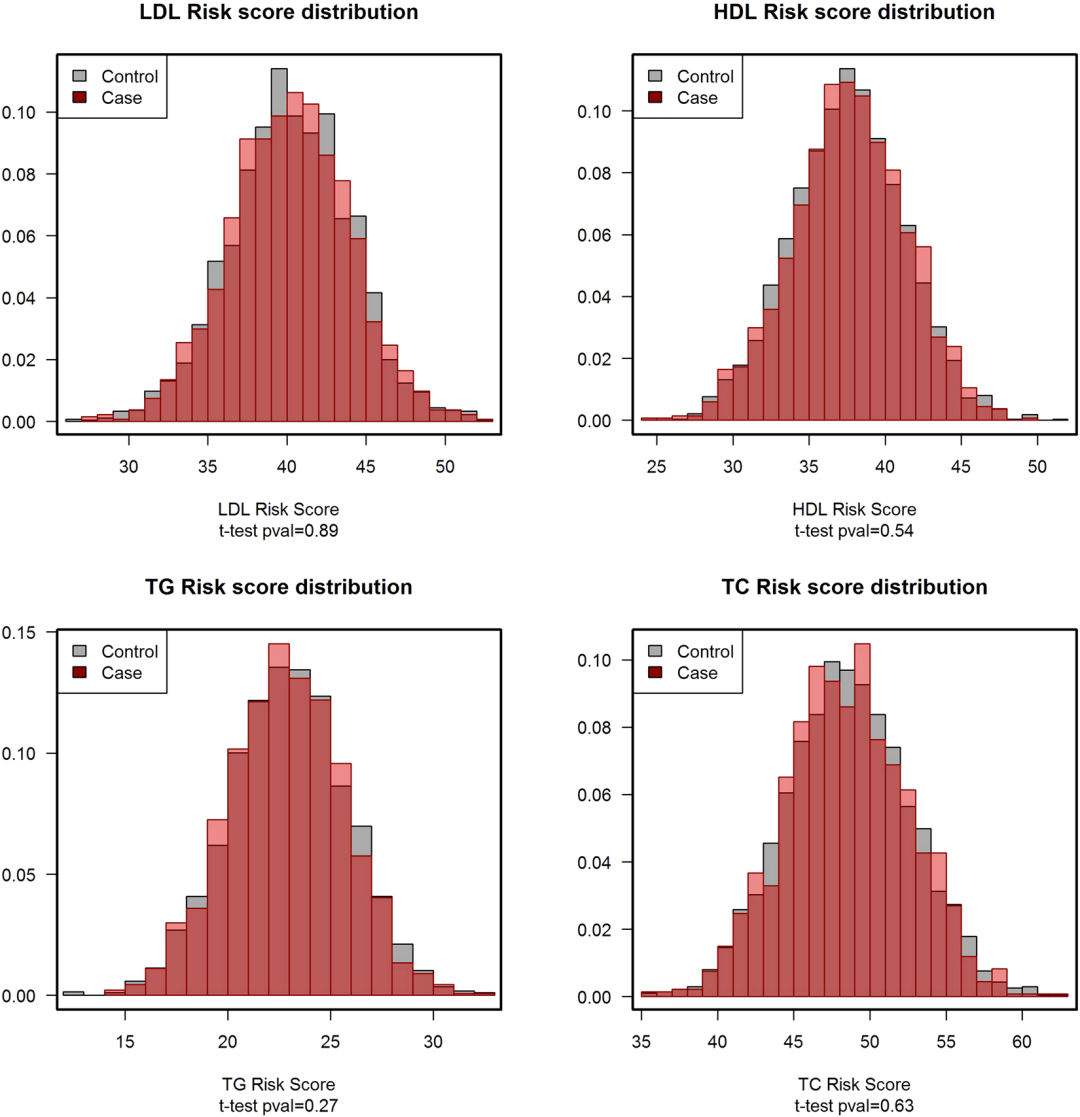
Distribution of the genetic risk score of every lipid trait in cases and controls. The x-axis indicates the number of risk alleles of the lipid genetic risk score indicated in each figure title. The y-axis corresponds to the proportion of subjects observed for each risk allele count. The proportion of controls are shown in gray and, superimposed, the proportion of cases in red. Deviations from the overlap between cases and controls are shown in light color. The distributions do not differ in shape or location as indicated in the t-tests performed to compare the number of risk alleles between cases and controls.

None of the Mendelian randomization estimates for the associations between LGS and CRC, based on unweighted allele scores, show any risk effect (Table 3). Cases had the same average risk alleles than controls in all the lipids traits (Table 3 and Fig. 2). Therefore, individuals with larger numbers of TG, HDL, LDL, or TC risk alleles were not at higher risk for CRC. Also, since the LDL and TC LGS were associated with statin use, we performed the analysis stratified by statin use. As Table 3 shows, the instruments were not associated with CRC among non-users of statins and there was not an effect interaction. Furthermore, to discard the possibility of confounding or pleiotropy, we confirmed that the LGS were independent of known CRC risk factors (Supplementary Table 5), and specifically discarded effect modification by BMI, diabetes or hypertension.

**Table 3:** Association of lipid genetic scores with CRC.

	Risk alleles in controls	Risk alleles in cases	OR^a^ per 10 risk alleles	95% CI	P-value
	Mean ± sd	Mean ± sd			
TG	23.44 ± 2.91	23.34 ± 2.82	0.91	0.72–1.16	0.44
HDL	38.05 ± 3.75	38.13 ± 3.75	1.14	0.95–1.37	0.16
LDL	40.71 ± 3.80	40.69 ± 3.80	0.97	0.81–1.16	0.73
TC	49.06 ± 4.11	49.00 ± 4.07	0.99	0.84–1.17	0.89
**Statins non-users**					
TG	23.40 ± 2.90	23.28 ± 2.83	0.90	0.69–1.18	0.46
HDL	38.04 ± 3.81	38.17 ± 3.72	1.20	0.98–1.47	0.076
LDL	40.50 ± 3.79	40.58 ± 3.75	1.06	0.87–1.30	0.56
TC	48.91 ± 4.08	48.86 ± 4.03	1.03	0.85–1.25	0.76
**Statins users**					
TG	23.62 ± 2.93	23.56 ± 2.74	0.99	0.58–1.71	0.98
HDL	38.13 ± 3.50	37.95 ± 3.92	0.91	0.59–1.39	0.64
LDL	41.57 ± 3.74	41.19 ± 3.97	0.74	0.49–1.12	0.15
TC	49.72 ± 4.17	49.59 ± 4.17	0.91	0.63–1.32	0.63

TG: triglycerides; HDL: high density lipoprotein cholesterol; LDL: low density lipoprotein cholesterol; TC: total cholesterol. All genetic scores are coded as increasing lipid levels.

^a^ORs and 95% CI derived from logistic regression models adjusted for the study design factors (age, sex, center and education). The quantitative genetic score calculated as the sum of risk alleles was divided by 10.

## Discussion

Mendelian randomization is a technique used to determine the causal impact of a risk factor on an outcome from observational data using genetic variants. It has already been used to investigate associations between blood lipids and colorectal polyps^27^, coronary heart disease^28^, and prostate cancer^29^. In our study, using the Mendelian randomization approach, none of the lipid genetic scores for dyslipidemia analyzed was associated with CRC risk. This indicates that lifetime dyslipidemia most probably is unrelated to the development of colorectal neoplasms, and that the associations previously found in observational studies between dyslipidemia and CRC could be result of uncontrolled confounding factors or reverse causation.

This analysis was based on the usual instrumental variable assumptions of Mendelian randomization studies that use genetic scores as lifetime exposure surrogates. The first assumption was that the LGS was associated with the exposure of interest. Our study could not test this condition directly since lipid levels were not measured, but it was probably fulfilled, since we selected SNPs that had been linked to plasma lipid levels in GWAS^20^. As an indirect check of this assumption, we confirmed the association between the TC, LDL and TG allele score and statin consumption, a drug that is essentially prescribed for high LDL but also for hypertriglyceridemia^30,31^. The second assumption was that the LGS was not related to other factors that confound the exposure-outcome relationship. Potential confounding factors (diabetes mellitus, arterial hypertension, BMI, and waist–hip ratio) that could influence the relationship between dyslipidemia and CRC were explored, and none were associated with the LGS. We acknowledge, however, that the existence of residual confounding cannot completely be ruled out in a case-control study. Since the genetic scores were related to statin use, we also performed an analysis stratified by use of these drugs and observed no effect modification. Mendelian randomization studies are usually robust to confounding, as long as the third assumption is met: the effect of the LGS on the outcome should be mediated only through the association with the modifiable risk factor. We cannot rule out pleiotropic effects of the SNPs included in the LGS that may confound the association, though we carefully selected the SNPs to avoid that. We excluded SNPs associated with other traits than the lipid of interest. In fact, these strong assumptions that Mendelian randomization studies require are suggested to be the reason why this type of study may find more negative results^22,23^.

It is uncertain whether dyslipidemia may contribute to the pathogenesis of colorectal neoplasia. In fact, three recent meta-analysis/systematic reviews show controversial results. Yao *et al*.^8^, concluded that especially high levels of TG and TC were associated with an increased risk of CRC, whereas HDL might be associated with a decreased risk of CRC. In contrast, Tian *et al*.^9^ reported that high levels of TC, TG and LDL were associated with colorectal adenoma but not with CRC, and no association was observed between levels HDL and colorectal neoplasia. Finally, Passarelli *et al*.^12^ found a 13% higher prevalence of colorectal adenomas per increase in TG levels at colonoscopy, a 4% lower per increase in HDL and similar blood concentrations of LDL and TC. There are two studies based on Mendelian randomization to assess the causality of dyslipidemia and colorectal neoplasia^11,27^. Passarelli *et al*.^27^ investigated the relationship between dyslipidemia and polyps and they reported that genotype–polyp odds ratio using weighted allele scores were not associated On the other hand, Rodriguez-Broadbent *et al*.^11^ supported a causal relationship between higher levels of TC with CRC risk. In our study, we did not find any association between dyslipidemia, including TC, and CRC. We have explored the relationship between TC and CRC and found it unrelated, though we believe its interpretation can be difficult as TC is essentially the sum of LDL and HDL and HDL may have an inverse effect on risk as it has been suggested for cardiovascular diseases^32^.

Regarding statins, these drugs have been suggested to play a role in cancer chemoprevention^33,34^. However, epidemiological evidence remains inconclusive. Recently, two meta-analyses including 40 and 42 individual studies, respectively^13,14^, reported a modest reduction in risk of CRC among statin users, though this was only among cohort studies and case control studies, and not among randomized controlled trials. It is unclear whether statin use may be associated with CRC due to their lipid lowering activity or to other mechanisms. Also, the association of statins with CRC could be just a reflection of the possible risk associated with high lipid levels. Confounding by indication is a common bias in observational studies and occurs when the indication (high cholesterol) for the medication under study (statin) is also associated with the outcome of interest (CRC). We have found that statin use was associated with CRC, with a borderline non-significant risk reduction of 16% in the multivariate adjusted model. However, we admit with a point estimate of 0.84 and a confidence interval which goes from 0.70 to 1.01, data must be interpreted cautiously. The failure to obtain a significant association could be related to statistical power, because our study is large, but not huge. We found, however, a stronger risk reduction for lipophilic statins. It is known that lipophilic statins show an efficient activity both at hepatic and extrahepatic sites, which could explain a higher effect in colonic tissue^35^. Since statins show a probable risk reduction of CRC but our Mendelian randomization study rules out the role of lipids, we should consider that the protective effect of statins is related to other mechanisms of action independent of lipid levels, such as anti-inflammatory effects^36^ or microbiome interactions^37^.

Although our study does not support a causal effect of dyslipidemia in CRC risk, we must consider that our study has several limitations. The strong assumptions required for Mendelian randomization analysis, as explained above, may not be fully met. Also, the modest sample size of our study results in a limited statistical power for a Mendelian randomization study unless the magnitude of the association is large^26,38^. Our calculations indicate that we have good power to detect OR > 1.33, assuming that the proportion of explained variance in lipid levels by the genetic score is 10%, which is debatable, but seems reasonable according to previous literature^39^. We assigned equal weight to each SNP, which may be suboptimal, but was necessary to include in the LGS some SNPs for which the effect size was unreported or were expressed in different units. Another important limitation is that we do not have serum lipid levels. However, the fact that the instrumental variable estimation for the LDL levels was associated with statins use supports the validity of the score used.

In conclusion, the null association observed in this Mendelian Randomization study does not support the hypothesis that dyslipidemia is involved in the pathogenesis of CRC. Our study found that lipophilic statins could have a possible protective effect on CRC by a mechanism unrelated to lipid levels.

## Supplementary information

Supplementary Tables

## Data Availability

The datasets generated and/or analyzed during the current study are not publicly available as they contain information that could compromise the privacy of research participants. The data are available from the corresponding author on request, but access to the data is dependent on consideration of the request by the Study Direction Committee, which serves as the data access committee for this study. The committee is affiliated to CIBER of Epidemiology and Public Health (CIBERESP), Instituto de Salud Carlos III, Madrid.
