# Supplementary material for: Mendelian randomization analysis rules out disylipidaemia as colorectal cancer cause
Source: Sci Rep. 2019 Sep 16;9:13407. doi: 10.1038/s41598-019-49880-w (PMC6746794; doi:10.1038/s41598-019-49880-w)
Supplement: Supplementary file 1 — Supplementary Tables [file 41598_2019_49880_MOESM1_ESM.doc]

**Mendelian randomization analysis rules out disylipidaemia as colorectal cancer cause.**

Gemma Ibáñez-Sanz^1,2,3^; Anna Díez-Villanueva^1^; Marina Riera-Ponsati^1^; Tania Fernández-Villa^4^; Pablo Fernández Navarro^3,5,6^; Mariona Bustamante^3,7,8,9^; Javier Llorca^3,10^; Pilar Amiano^3,11^; Nieves Ascunce^3,12,13^; Guillermo Fernández-Tardón^3,14^; Inmaculada Salcedo Bellido^3,15,16^; Dolores Salas^3,17,18^; Rocío Capelo Álvarez^19^; Marta Crous-Bou^1,20,21,22^; Luis Ortega-Valín^3,23^;Beatriz Pérez-Gómez^3,5,6^; Gemma Castaño-Vinyals^3,7,8,9^; Camilo Palazuelos^10^; Jone M Altzibar^11^; Eva Ardanaz^3,12,13^; Adonina Tardón^3,14^; José Juan Jiménez Moleón^3,15,16^; Valle Olmos Juste^24^; Nuria Aragonés^3,25^; Marina Pollán^3,5,6^; Manolis Kogevinas^3,7,8,9^; Victor Moreno^1,3,26*^

^1^Unit of Biomarkers and Susceptibility, Cancer Prevention and Control Program, Catalan Institute of Oncology (ICO) and ONCOBELL Program, Bellvitge Biomedical Research Institute (IDIBELL), L’Hospitalet de Llobregat, Spain

^2^Gastroenterology Department, Bellvitge University Hospital-IDIBELL, L’Hospitalet de Llobregat, Spain

^3^CIBER Epidemiología y Salud Pública (CIBERESP), Spain

^4^Grupo de Investigación en Interacciones Gen-Ambiente y Salud. Instituto de Biomedicina (IBIOMED). University of León. León, España

^5^Environmental and Cancer Epidemiology Department, National Center of Epidemiology - Instituto de Salud Carlos III, Madrid, Spain

^6^Oncology and Hematology Area, IIS Puerta de Hierro, Cancer Epidemiology Research Group, Madrid, Spain

^7^ISGlobal, Barcelona, Spain

^8^IMIM (Hospital del Mar Medical Research Institute), Barcelona, Spain

^9^ University of Pompeu Fabra, Barcelona, Spain

^10^ University of Cantabria – IDIVAL, Santander, Spain

^11^Public Health Division of Gipuzkoa, Biodonostia Research Institute, San Sebastian, Spain

^12^Navarra Public Health Institute, Pamplona, Spain

^13^IdiSNA, Navarra Institute for Health Research, Pamplona, Spain

^14^University Institute of Oncology of Asturias (IUOPA), Universidad de Oviedo, Oviedo, Spain

^15^Instituto de Investigación Biosanitaria de Granada (ibs.GRANADA), Hospitales Universitarios de Granada/ University of Granada, Granada, Spain

^16^Department of Preventive Medicine and Public Health, Faculty of Medicine, University of Granada, Granada, Spain

^17^Cancer and Public Health Area, FISABIO - Public Health, Valencia, Spain

^18^General Directorate Public Health, Valencia, Spain

^19^Centro de Investigación en Recursos Naturales, Salud, y Medio Ambiente (RENSMA), University of Huelva, Spain

^20^Barcelonaβeta Brain Research Center - Pasqual Maragall Foundation, Barcelona, Spain

^21^CIBER Fragilidad y Envejecimiento Saludable (CIBERFES), Madrid, Spain

^22^Department of Epidemiology, Harvard T. H. Chan School of Public Health, Boston, MA, USA

^23^Department of Pharmacy, Complejo Asistencial Universitario de León, Spain

^24^Department of General and Gastrointestinal Surgery, Complejo Asistencial Universitario de León, León, Spain

^25^Dirección General de Salud Pública, Consejería de Sanidad de la Comunidad de Madrid, Madrid, Spain

^26^Department of Clinical Sciences, Faculty of Medicine, University of Barcelona, Barcelona, Spain

**Supplementary Table 1**

Association of individual TG SNPs with CRC in the study population

| **SNP** | **Locus** | **Reported Gene** | **Risk Allele** | **Genotypes** | **Obs Genotype^1^** | **Exp Genotype^2^** | **OR MCC-Study** | **95% CI** | | | **P-value^3^** |
| --- | --- | --- | --- | --- | --- | --- | --- | --- | --- | --- | --- |
| rs1748195 | 1p31.3 | ANGPTL3 | C | 2096/1638/344 | 0.40 | 0.41 | 0.86 | 0.78 | - | 0.95 | 0.0034 |
| rs645040 | 3q22.3 | MSL2L1 | T | 2211/1578/291 | 0.39 | 0.39 | 1.03 | 0.93 | - | 1.15 | 0.55 |
| rs442177 | 4q22.1 | KLHL8 | T | 1856/1758/457 | 0.43 | 0.44 | 0.97 | 0.88 | - | 1.07 | 0.53 |
| rs9686661 | 5q11.2 | MAP3K1 | T | 232/1480/2368 | 0.36 | 0.36 | 0.90 | 0.80 | - | 1.00 | 0.05 |
| rs2247056 | 6p21.33 | HLA | C | 2958/1016/98 | 0.25 | 0.25 | 0.99 | 0.87 | - | 1.12 | 0.85 |
| rs38855 | 7q31.2 | MET | A | 1125/2031/897 | 0.50 | 0.50 | 1.03 | 0.94 | - | 1.13 | 0.53 |
| rs2240466 | 7q11.23 | MLXIPL | G | 3421/603/46 | 0.15 | 0.16 | 1.17 | 0.99 | - | 1.39 | 0.07 |
| rs11776767 | 8p23.1 | PINX1 | C | 687/1936/1411 | 0.48 | 0.48 | 0.94 | 0.86 | - | 1.03 | 0.20 |
| rs7819412 | 8p23.1 | XKR6 | A | 1176/2022/882 | 0.50 | 0.50 | 0.97 | 0.89 | - | 1.07 | 0.56 |
| rs1495741 | 8p22 | NAT2 | G | 205/1398/2477 | 0.34 | 0.34 | 1.00 | 0.89 | - | 1.12 | 1.00 |
| rs10096633 | 8p21.3 | LPL | C | 2798/1165/117 | 0.29 | 0.28 | 1.04 | 0.92 | - | 1.18 | 0.49 |
| rs2068888 | 10q23.33 | CYP26A1 | G | 1152/1982/946 | 0.49 | 0.50 | 0.97 | 0.88 | - | 1.06 | 0.49 |
| rs10761731 | 10q21.3 | JMJD1C | A | 1184/1969/927 | 0.48 | 0.50 | 1.03 | 0.94 | - | 1.13 | 0.48 |
| rs1832007 | 10p15.1 | AKR1C4 | A | 3164/856/60 | 0.21 | 0.21 | 0.87 | 0.75 | - | 1.00 | 0.05 |
| rs4938303 | 11q23.3 | ZNF259 | C | 310/1570/2194 | 0.39 | 0.39 | 0.92 | 0.83 | - | 1.02 | 0.11 |
| rs28927680 | 11q23.3 | APOA1 | G | 24/537/3519 | 0.13 | 0.13 | 0.92 | 0.77 | - | 1.11 | 0.38 |
| rs2929282 | 15q15.3 | FRMD5 | T | 14/477/3589 | 0.12 | 0.12 | 1.03 | 0.85 | - | 1.24 | 0.80 |
| rs2412710 | 15q15.1 | CAPN3 | A | 16/484/3580 | 0.12 | 0.12 | 1.09 | 0.90 | - | 1.31 | 0.38 |
| rs8077889 | 17q21.31 | MPP3 | C | 210/1468/2393 | 0.36 | 0.36 | 1.06 | 0.95 | - | 1.18 | 0.31 |
| rs7248104 | 19p13.2 | INSR | G | 1301/1986/793 | 0.49 | 0.49 | 1.03 | 0.94 | - | 1.13 | 0.52 |
| rs58542926 | 19p13.11 | CILP2 | C | 3616/450/13 | 0.11 | 0.11 | 1.06 | 0.87 | - | 1.30 | 0.55 |
| rs17216525 | 19p13.11 | NCAN | C | 3523/534/23 | 0.13 | 0.13 | 1.07 | 0.89 | - | 1.28 | 0.47 |
| rs1495741 | 8p22 | NAT2 | G | 205/1398/2477 | 0.34 | 0.34 | 1.00 | 0.89 | - | 1.12 | 1.00 |

^1^Observed genotypes in controls.

^2^Expected genotypes in controls

**^3^**Unadjusted P-values. Bonferroni significant if P-value < 0.002 (none significant)

**Supplementary Table 2**

Association of individual HDL SNPs with CRC in the study population

| **SNP** | **Locus** | **Reported Gene** | **Risk Allele** | **Genotypes** | **Obs Genotype^1^** | **Exp Genotype^2^** | **OR MCC-Study** | **95% CI** | | | **P-value^3^** |
| --- | --- | --- | --- | --- | --- | --- | --- | --- | --- | --- | --- |
| rs4660293 | 1p34.3 | PABPC4 | A | 2484/1363/233 | 0.33 | 0.35 | 1.08 | 0.97 | - | 1.21 | 0.18 |
| rs12145743 | 1q23.1 | HDGF | G | 508/1891/1681 | 0.46 | 0.46 | 1.07 | 0.97 | - | 1.18 | 0.17 |
| rs4650994 | 1q25.2 | ANGPTL1 | G | 1231/2007/842 | 0.49 | 0.5 | 1.03 | 0.94 | - | 1.13 | 0.55 |
| rs1689800 | 1q25.3 | ZNF648 | A | 1492/1932/656 | 0.47 | 0.48 | 1.00 | 0.91 | - | 1.10 | 0.95 |
| rs2144300 | 1q42.13 | GALNT2 | T | 1677/1875/528 | 0.46 | 0.46 | 1.07 | 0.97 | - | 1.18 | 0.17 |
| rs12328675 | 2q24.3 | COBLL1 | C | 94/1075/2908 | 0.26 | 0.26 | 0.94 | 0.83 | - | 1.07 | 0.38 |
| rs13326165 | 3p21.1 | STAB1 | A | 217/1397/2466 | 0.34 | 0.35 | 1.02 | 0.92 | - | 1.14 | 0.67 |
| rs2814944 | 6p21.31 | C6orf106 | G | 3041/966/72 | 0.24 | 0.24 | 0.99 | 0.87 | - | 1.14 | 0.91 |
| rs605066 | 6q24.1 | CITED2 | T | 1686/1870/509 | 0.46 | 0.46 | 1.08 | 0.98 | - | 1.19 | 0.12 |
| rs1084651 | 6q26 | LPA | G | 3076/922/81 | 0.23 | 0.23 | 1.02 | 0.89 | - | 1.17 | 0.79 |
| rs4917014 | 7p12.2 | IKZF1 | G | 527/1892/1661 | 0.46 | 0.46 | 0.93 | 0.84 | - | 1.02 | 0.14 |
| rs4731702 | 7q32.2 | KLF14 | T | 759/1921/1400 | 0.47 | 0.49 | 1.02 | 0.93 | - | 1.12 | 0.71 |
| rs2293889 | 8q23.3 | TRPS1 | G | 1489/1919/667 | 0.47 | 0.48 | 1.05 | 0.96 | - | 1.16 | 0.3 |
| rs581080 | 9p22.3 | TTC39B | C | 2562/1334/184 | 0.33 | 0.33 | 1.08 | 0.97 | - | 1.21 | 0.18 |
| rs3905000 | 9q31.1 | ABCA1 | G | 2752/1177/151 | 0.29 | 0.3 | 1.08 | 0.95 | - | 1.21 | 0.24 |
| rs970548 | 10q11.22 | MARCH8 | C | 308/1602/2170 | 0.39 | 0.4 | 0.93 | 0.84 | - | 1.04 | 0.2 |
| rs3136441 | 11p11.2 | LRP4 | C | 21/593/3466 | 0.15 | 0.14 | 1.06 | 0.89 | - | 1.25 | 0.53 |
| rs7395581 | 11p11.2 | MADD | G | 199/1429/2437 | 0.35 | 0.35 | 1.04 | 0.94 | - | 1.17 | 0.43 |
| rs7395662 | 11p11.2 | FOLH1 | A | 588/1865/1627 | 0.46 | 0.47 | 0.99 | 0.9 | - | 1.09 | 0.82 |
| rs12801636 | 11q13.1 | KAT5 | A | 226/1424/2430 | 0.35 | 0.35 | 1.00 | 0.9 | - | 1.12 | 0.99 |
| rs499974 | 11q13.5 | MOGAT2 | C | 2926/1058/96 | 0.26 | 0.26 | 0.92 | 0.81 | - | 1.05 | 0.22 |
| rs7941030 | 11q24.1 | UBASH3B | C | 487/1854/1737 | 0.45 | 0.45 | 0.89 | 0.81 | - | 0.98 | 0.02 |
| rs7134375 | 12p12.2 | PDE3A | A | 752/1975/1353 | 0.48 | 0.49 | 1.02 | 0.93 | - | 1.12 | 0.71 |
| rs7134594 | 12q24.11 | MVK | T | 1256/1983/841 | 0.49 | 0.49 | 0.95 | 0.87 | - | 1.04 | 0.28 |
| rs838880 | 12q24.31 | SCARB1 | C | 370/1668/2042 | 0.41 | 0.42 | 0.96 | 0.87 | - | 1.06 | 0.45 |
| rs4983559 | 14q32.33 | ZBTB42 | G | 623/1869/1588 | 0.46 | 0.47 | 1.01 | 0.92 | - | 1.11 | 0.85 |
| rs1800588 | 15q21.3 | LIPC | T | 212/1400/2466 | 0.34 | 0.35 | 1.02 | 0.91 | - | 1.14 | 0.78 |
| rs2652834 | 15q22.2 | LACTB | G | 2602/1326/152 | 0.32 | 0.32 | 1.04 | 0.93 | - | 1.17 | 0.49 |
| rs16942887 | 16q22.1 | LCAT | A | 88/1052/2939 | 0.26 | 0.26 | 1.01 | 0.89 | - | 1.15 | 0.89 |
| rs11869286 | 17q12 | STARD3 | C | 1590/1894/594 | 0.46 | 0.47 | 0.92 | 0.84 | - | 1.01 | 0.1 |
| rs4129767 | 17q25.3 | PGS1 | A | 945/2004/1131 | 0.49 | 0.5 | 0.96 | 0.88 | - | 1.05 | 0.39 |
| rs7241918 | 18q21.1 | LIPG | T | 2994/1003/81 | 0.25 | 0.24 | 1.02 | 0.9 | - | 1.17 | 0.72 |
| rs2967605 | 19p13.2 | ANGPTL4 | C | 2547/1364/169 | 0.33 | 0.33 | 1.01 | 0.91 | - | 1.14 | 0.8 |
| rs737337 | 19p13.2 | ANGPTL8 | T | 3485/570/23 | 0.14 | 0.14 | 1.07 | 0.9 | - | 1.28 | 0.44 |
| rs17695224 | 19q13.41 | HAS1 | G | 1931/1727/422 | 0.42 | 0.43 | 1.01 | 0.92 | - | 1.12 | 0.77 |
| rs181362 | 22q11.21 | UBE2L3 | C | 2729/1217/134 | 0.3 | 0.03 | 1.09 | 0.96 | - | 1.23 | 0.17 |
| rs581080 | 9p22.3 | TTC39B | C | 2562/1334/184 | 0.33 | 0.33 | 1.08 | 0.97 | - | 1.21 | 0.18 |
| rs970548 | 10q11.22 | MARCH8 | C | 308/1602/2170 | 0.39 | 0.4 | 0.93 | 0.84 | - | 1.04 | 0.2 |
| rs7941030 | 11q24.1 | UBASH3B | C | 487/1854/1737 | 0.45 | 0.45 | 0.89 | 0.81 | - | 0.98 | 0.02 |

^1^Observed genotypes in controls.

^2^Expected genotypes in controls

**^3^**Unadjusted P-values. Bonferroni significant if P-value < 0.002 (none significant)

**Supplementary Table 3**

Association of individual LDL SNPs with CRC in the study population

| **SNP** | **Locus** | **Reported Gene** | **Risk Allele** | **Genotypes** | **Obs Genotype^1^** | **Exp Genotype^2^** | **OR MCC-Study** | **95% CI** | | | **P-value^3^** |
| --- | --- | --- | --- | --- | --- | --- | --- | --- | --- | --- | --- |
| rs12027135 | 1p36.11 | LDLRAP1 | T | 1185/2025/860 | 0.50 | 0.50 | 0.97 | 0.88 | - | 1.06 | 0.52 |
| rs11206510 | 1p32.3 | PCSK9 | T | 2646/1267/166 | 0.31 | 0.32 | 0.92 | 0.82 | - | 1.04 | 0.19 |
| rs2479409 | 1p32.3 | PCSK9 | G | 492/1877/1711 | 0.46 | 0.46 | 0.93 | 0.84 | - | 1.02 | 0.14 |
| rs629301 | 1p13.3 | SORT1 | T | 2592/1322/166 | 0.32 | 0.32 | 0.99 | 0.88 | - | 1.11 | 0.89 |
| rs267733 | 1q21.3 | ANXA9 | A | 2958/1033/89 | 0.25 | 0.25 | 1.11 | 0.97 | - | 1.26 | 0.13 |
| rs515135 | 2p24.1 | APOB | C | 2638/1268/174 | 0.31 | 0.32 | 0.99 | 0.88 | - | 1.12 | 0.91 |
| rs6544713 | 2p21 | ABCG8 | T | 461/1886/1733 | 0.46 | 0.45 | 0.98 | 0.89 | - | 1.08 | 0.64 |
| rs10490626 | 2q14.1 | INSIG2 | G | 3581/484/14 | 0.12 | 0.12 | 0.92 | 0.77 | - | 1.12 | 0.41 |
| rs2030746 | 2q14.2 | LOC84931 | T | 741/2001/1338 | 0.49 | 0.49 | 0.98 | 0.89 | - | 1.08 | 0.65 |
| rs1250229 | 2q35 | FN1 | C | 2341/1486/245 | 0.36 | 0.37 | 1.05 | 0.95 | - | 1.18 | 0.34 |
| rs12916 | 5q13.3 | HMGCR | C | 713/1958/1408 | 0.48 | 0.49 | 0.89 | 0.81 | - | 0.98 | 0.02 |
| rs4530754 | 5q23.2 | CSNK1G3 | A | 1370/2002/708 | 0.49 | 0.49 | 0.97 | 0.88 | - | 1.06 | 0.51 |
| rs1501908 | 5q33.3 | TIMD4 | C | 1676/1862/538 | 0.46 | 0.46 | 1.00 | 0.91 | - | 1.10 | 0.98 |
| rs3757354 | 6p22.3 | MYLIP | C | 2753/1186/141 | 0.29 | 0.30 | 0.97 | 0.86 | - | 1.10 | 0.65 |
| rs2142672 | 6p22.3 | MYLIP | G | 2276/1502/294 | 0.37 | 0.38 | 0.99 | 0.89 | - | 1.10 | 0.84 |
| rs3177928 | 6p21.32 | HLA | A | 72/909/3006 | 0.23 | 0.23 | 1.02 | 0.89 | - | 1.17 | 0.82 |
| rs1564348 | 6q25.3 | LPA | C | 90/1018/2972 | 0.25 | 0.25 | 1.01 | 0.89 | - | 1.15 | 0.86 |
| rs12670798 | 7p15.3 | DNAH11 | C | 207/1390/2482 | 0.34 | 0.34 | 1.09 | 0.98 | - | 1.22 | 0.13 |
| rs2072183 | 7p13 | NPC1L1 | C | 204/1336/2540 | 0.33 | 0.34 | 0.97 | 0.87 | - | 1.09 | 0.60 |
| rs2126259 | 8p23.1 | PPP1R3B | C | 3473/581/24 | 0.14 | 0.14 | 1.08 | 0.91 | - | 1.29 | 0.37 |
| rs10102164 | 8q11.23 | SOX17 | A | 88/1034/2954 | 0.25 | 0.25 | 1.17 | 1.03 | - | 1.33 | 0.02 |
| rs2081687 | 8q12.1 | CYP7A1 | T | 544/1824/1712 | 0.45 | 0.46 | 1.00 | 0.91 | - | 1.10 | 0.94 |
| rs3780181 | 9p24.2 | VLDLR | A | 3559/506/15 | 0.12 | 0.12 | 1.21 | 1.00 | - | 1.47 | 0.05 |
| rs635634 | 9q34.2 | ABO | T | 246/1454/2380 | 0.36 | 0.36 | 1.00 | 0.89 | - | 1.11 | 0.95 |
| rs2255141 | 10q25.2 | GPAM | A | 411/1754/1890 | 0.43 | 0.43 | 0.98 | 0.89 | - | 1.08 | 0.70 |
| rs174570 | 11q12.2 | FADS2 | C | 3047/953/80 | 0.23 | 0.24 | 1.04 | 0.91 | - | 1.19 | 0.56 |
| rs11065987 | 12q24.12 | BRAP | A | 1405/2003/672 | 0.49 | 0.48 | 1.02 | 0.92 | - | 1.12 | 0.74 |
| rs1169288 | 12q24.31 | HNF1A | C | 412/1727/1927 | 0.42 | 0.43 | 1.01 | 0.92 | - | 1.12 | 0.81 |
| rs4942486 | 13q13.1 | BRCA2 | T | 1005/2034/1036 | 0.5 | 0.50 | 1.02 | 0.93 | - | 1.12 | 0.60 |
| rs8017377 | 14q12 | NYNRIN | A | 827/1973/1280 | 0.48 | 0.49 | 1.05 | 0.95 | - | 1.15 | 0.33 |
| rs314253 | 17p13.1 | DLG4 | T | 2005/1693/382 | 0.42 | 0.42 | 1.01 | 0.91 | - | 1.12 | 0.86 |
| rs1801689 | 17q24.2 | APOH | C | 23/536/3521 | 0.13 | 0.13 | 0.92 | 0.77 | - | 1.11 | 0.38 |
| rs2228671 | 19p13.2 | LDLR | C | 2962/1021/97 | 0.25 | 0.25 | 0.96 | 0.85 | - | 1.10 | 0.59 |
| rs157580 | 19q13.32 | APOE | A | 1531/1890/659 | 0.46 | 0.48 | 1.06 | 0.96 | - | 1.16 | 0.24 |
| rs364585 | 20p12.1 | SPTLC3 | G | 1734/1869/477 | 0.46 | 0.45 | 0.98 | 0.89 | - | 1.08 | 0.71 |
| rs6102059 | 20q12 | MAFB | C | 2200/1574/306 | 0.39 | 0.39 | 0.99 | 0.89 | - | 1.09 | 0.78 |
| rs6029526 | 20q12 | TOP1 | A | 903/2044/1132 | 0.50 | 0.50 | 0.99 | 0.90 | - | 1.08 | 0.78 |
| rs5763662 | 22q12.2 | MTMR3 | T | 0/108/3970 | 0.03 | 0.03 | 0.94 | 0.63 | - | 1.42 | 0.78 |
| rs4253772 | 22q13.31 | PPARA | T | 65/817/3198 | 0.20 | 0.21 | 0.96 | 0.83 | - | 1.11 | 0.55 |
| rs12027135 | 1p36.11 | LDLRAP1 | T | 1185/2025/860 | 0.50 | 0.50 | 0.97 | 0.88 | - | 1.06 | 0.52 |
| rs2479409 | 1p32.3 | PCSK9 | G | 492/1877/1711 | 0.46 | 0.46 | 0.93 | 0.84 | - | 1.02 | 0.14 |
| rs629301 | 1p13.3 | SORT1 | T | 2592/1322/166 | 0.32 | 0.32 | 0.99 | 0.88 | - | 1.11 | 0.89 |
| rs10490626 | 2q14.1 | INSIG2 | G | 3581/484/14 | 0.12 | 0.12 | 0.92 | 0.77 | - | 1.12 | 0.41 |
| rs12916 | 5q13.3 | HMGCR | C | 713/1958/1408 | 0.48 | 0.49 | 0.89 | 0.81 | - | 0.98 | 0.02 |
| rs4530754 | 5q23.2 | CSNK1G3 | A | 1370/2002/708 | 0.49 | 0.49 | 0.97 | 0.88 | - | 1.06 | 0.51 |
| rs3757354 | 6p22.3 | MYLIP | C | 2753/1186/141 | 0.29 | 0.30 | 0.97 | 0.86 | - | 1.10 | 0.65 |
| rs3177928 | 6p21.32 | HLA | A | 72/909/3006 | 0.23 | 0.23 | 1.02 | 0.89 | - | 1.17 | 0.82 |
| rs1564348 | 6q25.3 | LPA | C | 90/1018/2972 | 0.25 | 0.25 | 1.01 | 0.89 | - | 1.15 | 0.86 |
| rs12670798 | 7p15.3 | DNAH11 | C | 207/1390/2482 | 0.34 | 0.34 | 1.09 | 0.98 | - | 1.22 | 0.13 |
| rs2072183 | 7p13 | NPC1L1 | C | 204/1336/2540 | 0.33 | 0.34 | 0.97 | 0.87 | - | 1.09 | 0.60 |
| rs10102164 | 8q11.23 | SOX17 | A | 88/1034/2954 | 0.25 | 0.25 | 1.17 | 1.03 | - | 1.33 | 0.02 |
| rs2081687 | 8q12.1 | CYP7A1 | T | 544/1824/1712 | 0.45 | 0.46 | 1.00 | 0.91 | - | 1.10 | 0.94 |
| rs3780181 | 9p24.2 | VLDLR | A | 3559/506/15 | 0.12 | 0.12 | 1.21 | 1.00 | - | 1.47 | 0.05 |
| rs635634 | 9q34.2 | ABO | T | 246/1454/2380 | 0.36 | 0.36 | 1.00 | 0.89 | - | 1.11 | 0.95 |
| rs2255141 | 10q25.2 | GPAM | A | 411/1754/1890 | 0.43 | 0.43 | 0.98 | 0.89 | - | 1.08 | 0.70 |

^1^Observed genotypes in controls.

^2^Expected genotypes in controls

**^3^**Unadjusted P-values. Bonferroni significant if P-value < 0.002 (none significant)

**Supplementary Table 4**

Association of individual TC SNPs with CRC in the study population

| **SNP** | **Locus** | **Reported Gene** | **Risk Allele** | **Genotypes** | **Obs Genotype^1^** | **Exp Genotype^2^** | **OR MCC-Study** | **95% CI** | | | **P-value^3^** |
| --- | --- | --- | --- | --- | --- | --- | --- | --- | --- | --- | --- |
| rs1495741 | 8p22 | NAT2 | G | 205/1398/2477 | 0.34 | 0.34 | 1.00 | 0.89 | - | 1.12 | 100 |
| rs58542926 | 19p13.11 | CILP2 | C | 3616/450/13 | 0.11 | 0.11 | 1.06 | 0.87 | - | 1.30 | 0.55 |
| rs581080 | 9p22.3 | TTC39B | C | 2562/1334/184 | 0.33 | 0.33 | 1.08 | 0.97 | - | 1.21 | 0.18 |
| rs970548 | 10q11.22 | MARCH8 | C | 308/1602/2170 | 0.39 | 0.40 | 0.93 | 0.84 | - | 1.04 | 0.20 |
| rs7941030 | 11q24.1 | UBASH3B | C | 487/1854/1737 | 0.45 | 0.45 | 0.89 | 0.81 | - | 0.98 | 0.02 |
| rs7241918 | 18q21.1 | LIPG | G | 81/1003/2994 | 0.25 | 0.24 | 0.98 | 0.85 | - | 1.11 | 0.72 |
| rs12027135 | 1p36.11 | LDLRAP1 | T | 1185/2025/860 | 0.50 | 0.50 | 0.97 | 0.88 | - | 1.06 | 0.52 |
| rs2479409 | 1p32.3 | PCSK9 | G | 492/1877/1711 | 0.46 | 0.46 | 0.93 | 0.84 | - | 1.02 | 0.14 |
| rs629301 | 1p13.3 | SORT1 | T | 2592/1322/166 | 0.32 | 0.32 | 0.99 | 0.88 | - | 1.11 | 0.89 |
| rs10490626 | 2q14.1 | INSIG2 | A | 14/484/3581 | 0.12 | 0.12 | 1.08 | 0.90 | - | 1.31 | 0.41 |
| rs12916 | 5q13.3 | HMGCR | C | 713/1958/1408 | 0.48 | 0.49 | 0.89 | 0.81 | - | 0.98 | 0.02 |
| rs4530754 | 5q23.2 | CSNK1G3 | A | 1370/2002/708 | 0.49 | 0.49 | 0.97 | 0.88 | - | 1.06 | 0.51 |
| rs3757354 | 6p22.3 | MYLIP | C | 2753/1186/141 | 0.29 | 0.30 | 0.97 | 0.86 | - | 1.10 | 0.65 |
| rs3177928 | 6p21.32 | HLA | A | 72/909/3006 | 0.23 | 0.23 | 1.02 | 0.89 | - | 1.17 | 0.82 |
| rs1564348 | 6q25.3 | LPA | C | 90/1018/2972 | 0.25 | 0.25 | 1.01 | 0.89 | - | 1.15 | 0.86 |
| rs12670798 | 7p15.3 | DNAH11 | C | 207/1390/2482 | 0.34 | 0.34 | 1.09 | 0.98 | - | 1.22 | 0.13 |
| rs2072183 | 7p13 | NPC1L1 | C | 204/1336/2540 | 0.33 | 0.34 | 0.97 | 0.87 | - | 1.09 | 0.60 |
| rs10102164 | 8q11.23 | SOX17 | A | 88/1034/2954 | 0.25 | 0.25 | 1.17 | 1.03 | - | 1.33 | 0.02 |
| rs2081687 | 8q12.1 | CYP7A1 | T | 544/1824/1712 | 0.45 | 0.46 | 1.00 | 0.91 | - | 1.10 | 0.94 |
| rs3780181 | 9p24.2 | VLDLR | A | 3559/506/15 | 0.12 | 0.12 | 1.21 | 1.00 | - | 1.47 | 0.05 |
| rs635634 | 9q34.2 | ABO | T | 246/1454/2380 | 0.36 | 0.36 | 1.00 | 0.89 | - | 1.11 | 0.95 |
| rs2255141 | 10q25.2 | GPAM | A | 411/1754/1890 | 0.43 | 0.43 | 0.98 | 0.89 | - | 1.08 | 0.70 |
| rs11065987 | 12q24.12 | BRAP | A | 1405/2003/672 | 0.49 | 0.48 | 1.02 | 0.92 | - | 1.12 | 0.74 |
| rs1169288 | 12q24.31 | HNF1A | C | 412/1727/1927 | 0.42 | 0.43 | 1.01 | 0.92 | - | 1.12 | 0.81 |
| rs314253 | 17p13.1 | DLG4 | T | 2005/1693/382 | 0.42 | 0.42 | 1.01 | 0.91 | - | 1.12 | 0.86 |
| rs2228671 | 19p13.2 | LDLR | C | 2962/1021/97 | 0.25 | 0.25 | 0.96 | 0.85 | - | 1.10 | 0.59 |
| rs6029526 | 20q12 | TOP1 | A | 903/2044/1132 | 0.50 | 0.50 | 0.99 | 0.90 | - | 1.08 | 0.78 |
| rs4253772 | 22q13.31 | PPARA | T | 65/817/3198 | 0.20 | 0.21 | 0.96 | 0.83 | - | 1.11 | 0.55 |
| rs1077514 | 1p36.12 | ASAP3 | T | 3191/826/63 | 0.20 | 0.21 | 1.00 | 0.87 | - | 1.16 | 0.96 |
| rs12027135 | 1p36.11 | LDLRAP1 | T | 1185/2025/860 | 0.50 | 0.50 | 0.97 | 0.88 | - | 1.06 | 0.52 |
| rs2479409 | 1p32.3 | PCSK9 | G | 492/1877/1711 | 0.46 | 0.46 | 0.93 | 0.84 | - | 1.02 | 0.14 |
| rs2131925 | 1p31.3 | ANGPTL3 | T | 2098/1638/344 | 0.40 | 0.41 | 0.86 | 0.78 | - | 0.95 | 0.0038 |
| rs7515577 | 1p22.1 | EVI5 | A | 2578/1313/183 | 0.32 | 0.33 | 1.09 | 0.97 | - | 1.23 | 0.13 |
| rs629301 | 1p13.3 | SORT1 | T | 2592/1322/166 | 0.32 | 0.32 | 0.99 | 0.88 | - | 1.11 | 0.89 |
| rs10490626 | 2q14.1 | INSIG2 | A | 14/484/3581 | 0.12 | 0.12 | 1.08 | 0.90 | - | 1.31 | 0.41 |
| rs7570971 | 2q21.3 | RAB3GAP1 | A | 1339/1955/786 | 0.48 | 0.49 | 1.04 | 0.95 | - | 1.14 | 0.42 |
| rs2287623 | 2q31.1 | ABCB11 | G | 765/1983/1332 | 0.49 | 0.49 | 0.97 | 0.89 | - | 1.07 | 0.59 |
| rs11563251 | 2q37.1 | UGT1A1 | T | 58/865/3155 | 0.21 | 0.21 | 0.85 | 0.74 | - | 0.98 | 0.03 |
| rs13315871 | 3p14.3 | PXK | G | 3399/660/21 | 0.16 | 0.16 | 0.96 | 0.82 | - | 1.14 | 0.67 |
| rs6831256 | 4p16.3 | LRPAP1 | G | 806/2033/1240 | 0.50 | 0.49 | 0.94 | 0.86 | - | 1.03 | 0.18 |
| rs12916 | 5q13.3 | HMGCR | C | 713/1958/1408 | 0.48 | 0.49 | 0.89 | 0.81 | - | 0.98 | 0.02 |
| rs4530754 | 5q23.2 | CSNK1G3 | A | 1370/2002/708 | 0.49 | 0.49 | 0.97 | 0.88 | - | 1.06 | 0.51 |
| rs6882076 | 5q33.3 | TIMD4 | C | 1674/1865/537 | 0.46 | 0.46 | 1.00 | 0.91 | - | 1.10 | 0.98 |
| rs3757354 | 6p22.3 | MYLIP | C | 2753/1186/141 | 0.29 | 0.30 | 0.97 | 0.86 | - | 1.10 | 0.65 |
| rs3177928 | 6p21.32 | HLA | A | 72/909/3006 | 0.23 | 0.23 | 1.02 | 0.89 | - | 1.17 | 0.82 |
| rs2814982 | 6p21.31 | C6orf106 | C | 3443/604/33 | 0.15 | 0.15 | 1.00 | 0.85 | - | 1.19 | 0.97 |
| rs9376090 | 6q23.3 | HBS1L | T | 2579/1304/179 | 0.32 | 0.33 | 0.95 | 0.84 | - | 1.06 | 0.34 |
| rs12208357 | 6q25.3 | LPA | T | 7/379/3661 | 0.09 | 0.09 | 1.17 | 0.95 | - | 1.45 | 0.14 |
| rs1564348 | 6q25.3 | LPA | C | 90/1018/2972 | 0.25 | 0.25 | 1.01 | 0.89 | - | 1.15 | 0.86 |
| rs1997243 | 7p22.3 | GPR146 | G | 97/951/3032 | 0.23 | 0.24 | 1.24 | 1.08 | - | 1.41 | 0.0013 |
| rs12670798 | 7p15.3 | DNAH11 | C | 207/1390/2482 | 0.34 | 0.34 | 1.09 | 0.98 | - | 1.22 | 0.13 |
| rs4722551 | 7p15.2 | MIR148A | C | 96/1066/2918 | 0.26 | 0.26 | 1.07 | 0.94 | - | 1.22 | 0.29 |
| rs2072183 | 7p13 | NPC1L1 | C | 204/1336/2540 | 0.33 | 0.34 | 0.97 | 0.87 | - | 1.09 | 0.60 |
| rs1495741 | 8p22 | NAT2 | G | 205/1398/2477 | 0.34 | 0.34 | 1.00 | 0.89 | - | 1.12 | 1.00 |
| rs10102164 | 8q11.23 | SOX17 | A | 88/1034/2954 | 0.25 | 0.25 | 1.17 | 1.03 | - | 1.33 | 0.02 |
| rs2081687 | 8q12.1 | CYP7A1 | T | 544/1824/1712 | 0.45 | 0.46 | 1.00 | 0.91 | - | 1.10 | 0.94 |
| rs2737229 | 8q23.3 | TRPS1 | A | 2379/1452/249 | 0.36 | 0.36 | 1.03 | 0.92 | - | 1.15 | 0.60 |
| rs3780181 | 9p24.2 | VLDLR | A | 3559/506/15 | 0.12 | 0.12 | 1.21 | 1.00 | - | 1.47 | 0.05 |
| rs581080 | 9p22.3 | TTC39B | C | 2562/1334/184 | 0.33 | 0.33 | 1.08 | 0.97 | - | 1.21 | 0.18 |
| rs635634 | 9q34.2 | ABO | T | 246/1454/2380 | 0.36 | 0.36 | 1.00 | 0.89 | - | 1.11 | 0.95 |
| rs10904908 | 10p13 | VIM | G | 839/2014/1227 | 0.49 | 0.50 | 0.94 | 0.85 | - | 1.03 | 0.17 |
| rs970548 | 10q11.22 | MARCH8 | C | 308/1602/2170 | 0.39 | 0.40 | 0.93 | 0.84 | - | 1.04 | 0.20 |
| rs2255141 | 10q25.2 | GPAM | A | 411/1754/1890 | 0.43 | 0.43 | 0.98 | 0.89 | - | 1.08 | 0.70 |
| rs10128711 | 11p15.1 | SPTY2D1 | C | 2282/1539/258 | 0.38 | 0.38 | 0.96 | 0.87 | - | 1.07 | 0.50 |
| rs174546 | 11q12.2 | FADS1 | C | 1998/1684/398 | 0.41 | 0.42 | 1.11 | 1.00 | - | 1.23 | 0.04 |
| rs11603023 | 11q23.3 | PHLDB1 | T | 817/1995/1268 | 0.49 | 0.49 | 1.06 | 0.97 | - | 1.17 | 0.19 |
| rs7941030 | 11q24.1 | UBASH3B | C | 487/1854/1737 | 0.45 | 0.45 | 0.89 | 0.81 | - | 0.98 | 0.02 |

^1^Observed genotypes in controls.

^2^Expected genotypes in controls

**^3^**Unadjusted P-values. Bonferroni significant if P-value < 0.002 (none significant)

**Supplementary Table 5**

Association of genetic scores and risk factors for CRC

| **Characteristic** | **TG** | | | **HDL** | | | **LDL** | | | **TC** | | |
| --- | --- | --- | --- | --- | --- | --- | --- | --- | --- | --- | --- | --- |
|  | **Beta** | **Standard error** | **P- value** | **Beta** | **Standard error** | **P- value** | **Beta** | **Standard error** | **P- value** | **Beta** | **Standard error** | **P- value** |
| **Age (years) at index date** | -0.007 | 0.005 | 0.14 | 0.001 | 0.006 | 0.86 | 0.005 | 0.006 | 0.42 | -0.001 | 0.006 | 0.87 |
| **Male sex** | 0.14 | 0.10 | 0.14 | 0.11 | 0.13 | 0.37 | 0.15 | 0.13 | 0.23 | 0.09 | 0.14 | 0.51 |
| **Family history of CRC** | 0.27 | 0.13 | 0.03 | 0.11 | 0.13 | 0.49 | 0.04 | 0.17 | 0.79 | 0.09 | 0.18 | 0.61 |
| **Cigarette smoking history** | 0.02 | 0.09 | 0.80 | 0.05 | 0.12 | 0.68 | -0.07 | 0.12 | 0.56 | -0.12 | 0.13 | 0.36 |
| **High risk consumption alcohol** | 0.08 | 0.12 | 0.48 | -0.18 | 0.15 | 0.24 | 0.048 | 0.16 | 0.76 | 0.08 | 0.17 | 0.66 |
| **Diabetes mellitus** | 0.14 | 0.13 | 0.29 | -0.04 | 0.16 | 0.81 | 0.27 | 0.17 | 0.10 | 0.08 | 0.18 | 0.66 |
| **Arterial hypertension** | 0.05 | 0.09 | 0.63 | -0.22 | 0.12 | 0.08 | -0.008 | 0.12 | 0.95 | -0.08 | 0.13 | 0.56 |
| **High waist–hip ratio^a^** | 0.12 | 0.11 | 0.28 | 0.13 | 0.14 | 0.36 | -0.01 | 0.14 | 0.93 | 0.20 | 0.15 | 0.18 |
| **Obesity (BMI ≥30 kg/m^2^)** | 0.18 | 0.17 | 0.27 | -0.03 | 0.22 | 0.91 | 0.30 | 0.22 | 0.17 | 0.12 | 0.24 | 0.53 |
| **Physical activity in leisure time** | 0.010 | 0.09 | 0.91 | 0.08 | 0.12 | 0.52 | -0.06 | 0.12 | 0.62 | 0.14 | 0.13 | 0.28 |
| **High intake of vegetables (> 200g/day)** | 0.001 | 0.10 | 1.00 | 0.005 | 0.13 | 0.97 | 0.21 | 0.13 | 0.11 | -0.03 | 0.14 | 0.80 |
| **High intake of red meat (> 65g/day)** | 0.002 | 0.09 | 0.98 | -0.09 | 0.12 | 0.43 | 0.16 | 0.12 | 0.19 | -0.13 | 0.13 | 0.33 |
| **Regular ASA users** | -0.09 | 0.14 | 0.53 | -0.23 | 0.18 | 0.21 | 0.18 | 0.19 | 0.33 | 0.23 | 0.20 | 0.23 |
| **Regular NSAIDs non-ASA users** | 0.02 | 0.12 | 0.84 | 0.03 | 0.16 | 0.84 | -0.16 | 0.16 | 0.34 | -0.01 | 0.18 | 0.95 |
| **Regular statin users** | 0.26 | 0.11 | 0.03 | 0.03 | 0.15 | 0.85 | 0.92 | 0.15 | <0.001 | 0.81 | 0.16 | <0.001 |
| **Stratified analysis** | **OR^b^** | **95% CI** | **P-value** | **OR^b^** | **95% CI** | **P-value** | **OR^b^** | **95% CI** | **P-value** | **OR^b^** | **95% CI** | **P-value** |
| **Diabetes mellitus Yes** | 0.95 | 0.90-1.01 | 0.11 | 1.04 | 0.99-1.08 | 0.13 | 0.99 | 0.95-1.04 | 0.78 | 1.00 | 0.95-1.03 | 0.51 |
| **Diabetes mellitus No** | 0.99 | 0.97-1.02 | 0.87 | 1.01 | 0.99-1.03 | 0.39 | 1.00 | 0.98-1.02 | 0.82 | 1.00 | 0.98-1.02 | 0.88 |
| **Arterial hypertension Yes** | 0.99 | 0.96-1.03 | 0.65 | 1.02 | 1.00-1.05 | 0.11 | 1.00 | 0.97-1.03 | 0.85 | 1.00 | 0.97-1.03 | 0.96 |
| **Arterial hypertension No** | 0.99 | 0.96-1.02 | 0.54 | 1.01 | 0.98-1.03 | 0.64 | 1.00 | 0.94-1.02 | 0.78 | 1.00 | 0.98-1.02 | 0.88 |
| **Obesity (BMI ≥30 kg/m^2^)** | 1.00 | 0.92-1.08 | 0.93 | 1.01 | 0.99-1.03 | 0.17 | 1.00 | 0.94-1.01 | 0.94 | 1.00 | 0.95-1.06 | 0.92 |
| **Obesity (BMI <30 kg/m^2^)** | 0.99 | 0.97-1.01 | 0.40 | 1.00 | 0.96-1.05 | 0.87 | 1.00 | 0.98-1.02 | 0.66 | 1.00 | 0.98-1.02 | 0.84 |

^a^ ≥0.90cm (men); ≥0.85cm (women)

^b^ ORs and 95% CI derived from logistic regression models adjusted for the study design factors (age, sex, center and education).
